# Involvement of the Autophagy Protein Atg1 in Development and Virulence in *Botryosphaeria dothidea*

**DOI:** 10.3390/jof8090904

**Published:** 2022-08-26

**Authors:** Na Liu, Meiqi Zhu, Yihan Zhang, Zhongqiang Wang, Baohua Li, Weichao Ren

**Affiliations:** College of Plant Health and Medicine, Qingdao Agricultural University, Qingdao 266109, China

**Keywords:** *Botryosphaeria dothidea*, autophagy, BdAtg1, development, virulence

## Abstract

Botryosphaeria canker and fruit rot caused by the fungus *Botryosphaeria dothidea* is one of the most destructive diseases of apple worldwide. Autophagy is an evolutionarily conserved self-degradation process that is important for maintaining homeostasis to ensure cellular functionality. To date, the role of autophagy in *B. dothidea* is not well elucidated. In this study, we identified and characterized the autophagy-related protein Atg1 in *B. dothidea*. The BdAtg1 deletion mutant Δ*BdAtg1* showed autophagy blockade and phenotypic defects in mycelial growth, conidiation, ascosporulation and virulence. In addition, Δ*BdAtg1* exhibited an increased number of nuclei in the mycelial compartment. Comparative transcriptome analysis revealed that inactivation of BdAtg1 significantly influenced multiple metabolic pathways. Taken together, our results indicate that BdAtg1 plays an important role in vegetative differentiation and the pathogenicity of *B. dothidea*. The results of this study will provide a reference for the development of new target-based fungicides.

## 1. Introduction

Botryosphaeria canker and fruit ring rot caused by the ascomycetous fungus *Botryosphaeria dothidea* (anamorph *Dothiorella gregaria*) is one of the most devastating diseases of apple worldwide [1,2]. The pathogen can infect both branches and fruits, and can occur in both pre- and post-harvesting phases, causing huge losses to apple production [3]. Currently, almost all apple varieties are susceptible to *B. dothidea*, and chemical control is still the most effective disease control strategy [4]. However, with the long-term and frequent application of chemical agents, *B. dothidea* populations have developed resistance to some fungicides [5]. Therefore, exploring the molecular mechanisms underlying development and virulence will contribute to disease resistance breeding and novel fungicide development, establishing a more efficient disease management strategy.

Over the past two decades, autophagy has attracted considerable attention as a conserved self-digestion process that is essential for the maintenance of cellular metabolism and energy homeostasis in eukaryotes [6,7]. The process of autophagy consists of several sequential steps: initiation, elongation, maturation, fusion and degradation, which was tightly regulated by a series of key components, including the family of autophagy-related genes (ATG) [8,9]. The Atg1/ULK1 plays a central role in the initiation of autophagy process: receiving signals of nutritional status, recruiting downstream Atg proteins to the autophagosome formation site, and governing the formation of autophagosome [10,11,12].

Studies on biological functions in eukaryotes from yeast to mammals have shown that Atg1 plays important roles in cellular development and differentiation [13]. Atg1 has been reported to be involved in mycelial growth, asexual and sexual reproduction, stress tolerance, secondary metabolism and pathogenicity in filamentous fungi such as *Magnaporthe oryzae*, *Botrytis cinerea* and *Fusarium graminearum* [14,15,16]. Despite the knowledge of autophagy and Atg1 in some fungi, the specific role of Atg1 in *B. dothidea* remains unknown until now. In this study, we identified and characterized BdAtg1 in *B. dothidea*, and determined its role in autophagy, fungal development and virulence.

## 2. Materials and Methods

### 2.1. Fungal Strains and Culture Conditions

The wild-type strain LW03 and mutants of *B. dothidea* were cultured on potato dextrose agar (PDA, 200 g potato, 20 g dextrose, 10 g agar and 1 L H_2_O) and minimal medium (MM, 0.5 g KCl, 2 g NaNO_3_, 1 g KH_2_PO_4_, 0.5 g MgSO_4_·7H_2_O, 0.01 g FeSO_4_·7H_2_O, 30 g sucrose, 200 μL trace element, 15 g agar and 1 L ddH_2_O, pH = 6.9) at 25 °C for mycelial growth test. Carrot agar medium (200 g carrot, 15 g agar and 1 L ddH_2_O) was used for conidial production. One-year-old branches of ‘Fuji’ apple were used for perithecial formation. Mycelia used for protein extraction were grown in yeast extract peptone dextrose medium (YEPD, 10 g peptone, 3 g yeast extract, 20 g dextrose and 1 L ddH_2_O, pH = 6.7) or MM-N (MM without nitrogen source).

### 2.2. Gene Deletion and Complementation

The *BdATG1* deletion constructs were generated using a double-joint PCR approach [17]. Targeted deletion of *BdATG1* was conducted based on a homologous recombination strategy (Figure S1A). The putative deletion mutants with hygromycin resistance (100 µg/mL) were preliminarily screened by PCR amplification and further identified by Southern blot hybridization analysis.

For complementation, the fragment containing the full-length of *BdATG1* and native promoter was inserted into PYF11 vector and transformed into the *BdATG1* deletion mutant. The expression of *BdATG1* in a complemented strain was detected by Western blotting assay. Protoplast preparation and transformation were performed as described previously [18].

### 2.3. Staining and Microscopy

The fresh mycelia of *B. dothidea* were stained with CMAC (7-amino-4-chloromethylcoumarin) and BODIPY (dipyrrometheneboron difluoride) to observe vacuoles and lipid droplets, respectively. Microscopic examination was performed using a Leica TCS SP5 confocal laser scanning microscope (Leica, Wetzlar, Germany).

### 2.4. Protein Manipulation and Western Blotting

Mycelia for protein extraction were grown in a liquid medium at 25 °C with 180-rpm rotation. The total protein was extracted from mycelia as described previously [19]. A monoclonal anti-GFP antibody (ab 32146, Abcam, Cambridge, MA, USA) and an anti-glyceraldehyde-3-phosphate dehydrogenase (GAPDH) antibody (EM1101, Hangzhou Huaan Biotechnology Co., Ltd., Hangzhou, China) were used for immunoblot analyses. These experiments were repeated three times independently.

### 2.5. Pathogenicity Tests

Pathogenicity of *B. dothidea* was determined by inoculating detached fruits, branches and leaves of ‘Fuji’ apple. Briefly, the samples were wounded and inoculated with agar plugs of actively growing mycelia. Agar plugs without mycelia were used as a negative control. The inoculated samples were placed at 25 °C under high relative humidity (~95%) conditions. The disease lesions developed on inoculated samples were measured and photographed at 7 days post-inoculation. These experiments were repeated three times with 10 replicates in each repeat experiment.

### 2.6. Transcriptome Sequencing and Analysis

Total RNA was isolated using Trizol reagent (Takara Inc., Dalian, China), according to the manufacturer’s instructions. The sequencing libraries were generated using NEBNext UltraTM RNA Library Prep Kit for Illumina (NEB, Ipswich, MA, USA), following the manufacturer’s recommendations. The clustering of the index-coded samples was performed on a cBot Cluster Generation System using TruSeq Cluster Kit v3-cBot-HS (Illumia, Bologna, Italy), according to the manufacturer’s instructions. The library preparations were sequenced on an Illumina Nova platform and 150 bp paired-end reads were generated. Differential expression analysis was performed using DESeq2 R package (1.16.1). The ClusterProfiler R package was used to perform the statistical enrichment of differential expression genes in KEGG pathways and Gene Ontology (GO).

## 3. Results

### 3.1. Identification of BdATG1 in B. dothidea

**Figure 1:**
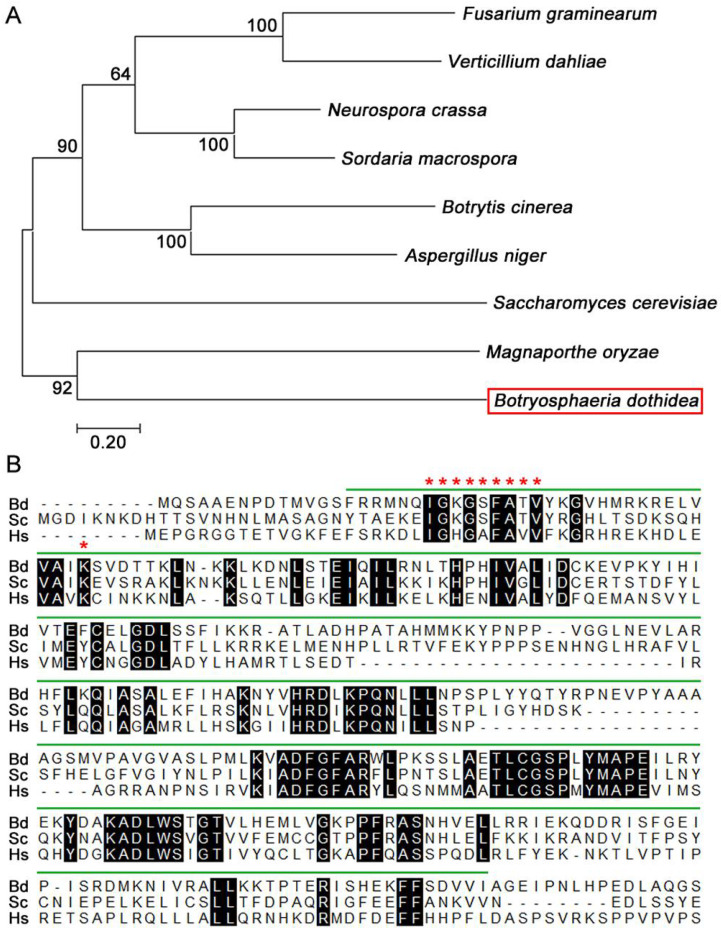
Evolutionary analysis of BdAtg1. (**A**) Phylogenetic tree of BdAtg1 and other orthologs from fungi, including *Fusarium graminearum*, *Verticillium dahliae*, *Neurospora crassa*, *Sordaria macrospora*, *Botrytis cinerea*, *Aspergillus niger*, *Magnaporthe oryzae* and *Saccharomyces cerevisiae*. (**B**) Sequence alignment of serine-threonine kinase domains form *B. dothidea*, S. *cerevisiae* and *Humo sapiens*. The green overlines indicate the serine-threonine kinase domain and the red asterisks indicate ATP-binding sites.

To obtain the Atg1 encoding gene in *B. dothidea*, the protein sequence of Atg1 from *Saccharomyces cerevisiae* was used to perform a BLASTp search in the genome database of *B. dothidea* (https://www.ncbi.nlm.nih.gov/biosample/SAMN13735646, accessed on 19, March, 2020). *BdATG1* is 3049 bp in length with 2 introns, and is predicted to encode a protein of 966 amino acids which shares 47.09% identity with *S. cerevisiae* Atg1. Phylogenetic analysis revealed that BdAtg1 shared a close ancestor with Atg1 of *M. oryzae* (Figure 1A). Sequence alignment revealed that the serine/threonine kinase domain of Atg1 is evolutionarily conserved among *B. dothidea*, *S. cerevisiae* and *H. sapiens* (Figure 1B).

### 3.2. Deletion and Complementation of BdATG1 in B. dothidea

To investigate the biological functions of *BdATG1*, we generated *BdATG1* deletion mutants by using a homologous recombination strategy (Figure S1A). The putative transformants were first screened by PCR amplification (Figure S1B) with diagnostic primers (Table S1), and further identified by southern blot analysis (Figure S1C). To complement the *BdATG1* deletion mutant, the full-length *BdATG1* (under native promoter) was transformed into Δ*BdAtg1*, and the expression of *BdATG1* was detected by Western blotting assay (Figure S1D).

### 3.3. BdAtg1 Is Indispensable for Autophagy

**Figure 2:**
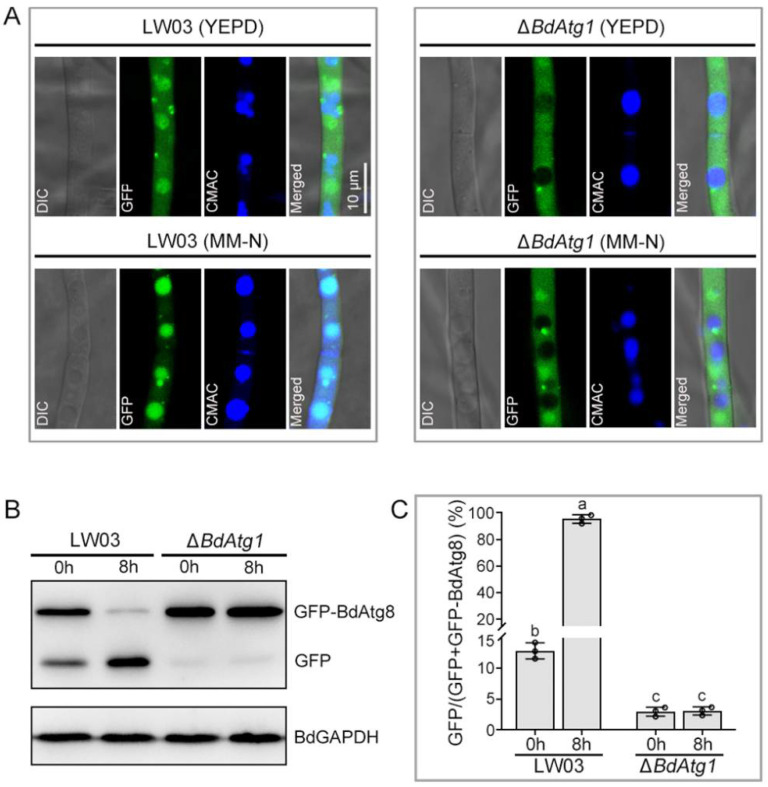
BdAtg1 is indispensable for autophagy. (**A**) GFP-BdAtg8 localization in the mycelia of the wild-type strain LW03 and *BdATG1* deletion mutant Δ*BdAtg1* under the conditions of sufficient or lack of nitrogen source. (**B**) GFP-BdAtg8 proteolysis of each strain (starvation for 0 and 8 h) was detected by Western blotting using anti-GFP antibody. GAPDH was used as an internal reference. (**C**) The percentage of GFP on the total of GFP and GFP-BdAtg8. Error bars indicate standard deviation from three independent experiments. Values on the bars followed by the same letter are not significantly different at *p* = 0.05.

GFP-Atg8 is a useable marker to monitor autophagy process [20]. To clarify the effect of BdAtg1 on autophagy, we examined the occurrence of autophagy in LW03 and Δ*BdAtg1*. As shown in Figure 2A, the GFP-BdAtg8 signals of LW03 transferred to vacuoles under nitrogen-lacking conditions, however, no GFP-BdAtg8 signals were observed in the vacuoles of Δ*BdAtg1*. In addition, the proteolysis of GFP-BdAtg8 was analyzed. The protein levels of full-length GFP-BdAtg8 were significantly decreased in LW03 but not in Δ*BdAtg1* under nitrogen-lacking conditions (Figure 2B,C). These results indicate that BaAtg1 is essential for autophagy in *B. dothidea*.

### 3.4. BdAtg1 Is Involved in Vegetative Growth

**Figure 3:**
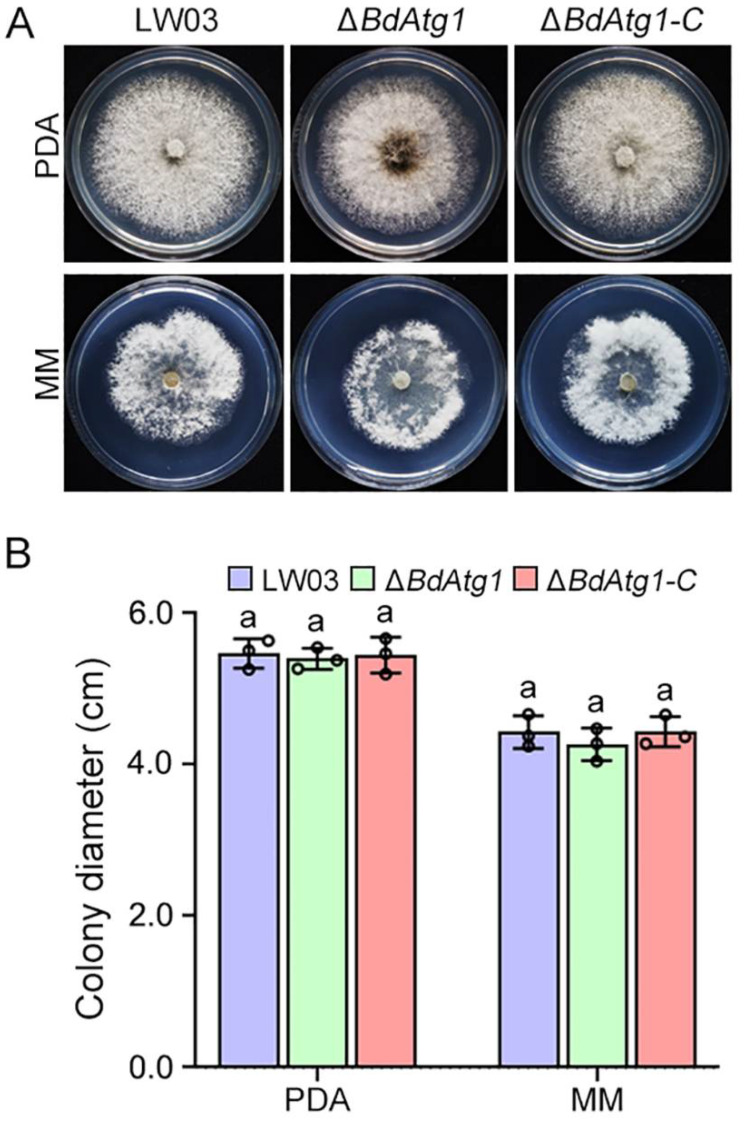
BdAtg1 is involved in mycelial growth. (**A**) Strains of *B. dothidea* were grown on PDA and MM media at 25 °C for 3 days. (**B**) Mycelial growth rate of each strain on PDA and MM media. Error bars indicate standard deviation from three independent experiments. Values on the bars followed by the same letter are not significantly different at *p* = 0.05.

To determine the role of BdAtg1 in mycelial growth, the wild-type strain LW03, *BdATG1* deletion mutant Δ*BdAtg1* and the complemented strain Δ*BdAtg1-C* were cultured on PDA and MM media. Compared with LW03 and Δ*BdAtg1-C*, the amount of aerial mycelium in Δ*BdAtg1* was significantly reduced (Figure 3A), but the growth rate showed no significant change (Figure 3B). These results indicate that BdAtg1 is involved in vegetative growth in *B. dothidea*.

### 3.5. BdAtg1 Is Crucial for Asexual and Sexual Reproduction

**Figure 4:**
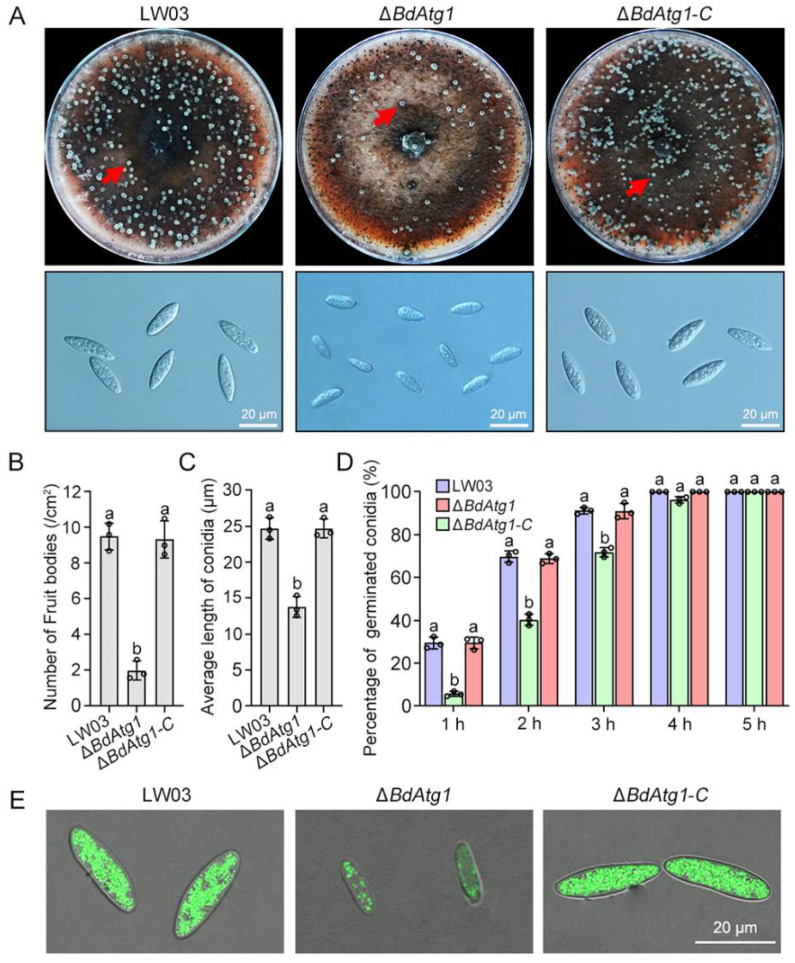
BdAtg1 is crucial for conidiation. (**A**) Fruiting bodies formed on carrot agar medium by *B. dothidea* strains (upper panel), and conidial morphology of each strain (bottom panel). The red arrows in indicate fruiting bodies. (**B**) Number of fruiting bodies formed by each strain. (**C**) Average length of conidia of each strain. (**D**) Conidia germination rete of each strain. Error bars indicate standard deviation from three independent experiments. Values on the bars followed by the same letter are not significantly different at *p* = 0.05. (**E**) Lipid droplets within conidia of each strain.

To determine the role of BdAtg1 in conidiation, each strain was cultured on carrot agar medium. After 7 days of incubation with black light exposure at 25 °C, LW03 and Δ*BdAtg1-C* produced many fruiting bodies containing conidia, while Δ*BdAtg1* produced fewer fruiting bodies containing malformed conidia (Figure 4A,B). The average length of the conidia of Δ*BdAtg1* was significantly lower than that of LW03 and Δ*BdAtg1-C* (Figure 4C), and the conidia germination of Δ*BdAtg1* was delayed (Figure 4D). In addition, a significant decrease of lipid droplets was observed in Δ*BdAtg1* compared to LW03 and Δ*BdAtg1-C* (Figure 4E).

**Figure 5:**
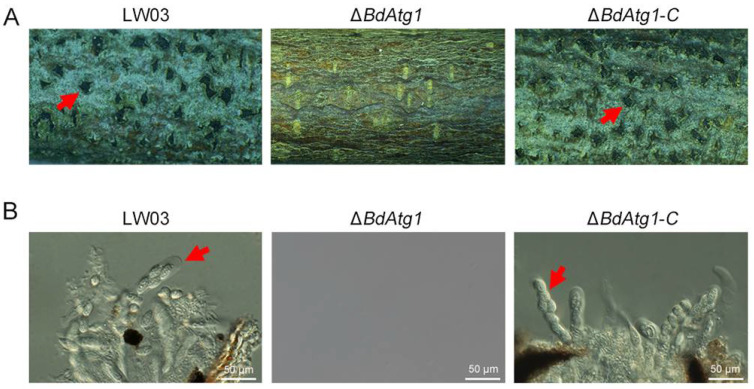
BdAtg1 is essential for ascosporulation. (**A**) Perithecia formed on apple branches by *B. dothidea* strains. The red arrows indicate asci. (**B**) Ascus and ascospores produced by each strain.

To determine the role of BdAtg1 in ascosporulation, each strain was inoculated to one-year-old apple branches. After 30 days of incubation at room temperature with intermittent water spray, Δ*BdAtg1* failed to form ascus, while LW03 and Δ*BdAtg1-C* formed many asci containing ascospores (Figure 5). These results indicate that BdAtg1 plays an important role in asexual and sexual reproduction.

### 3.6. BdAtg1 Is Required for Full Virulence

**Figure 6:**
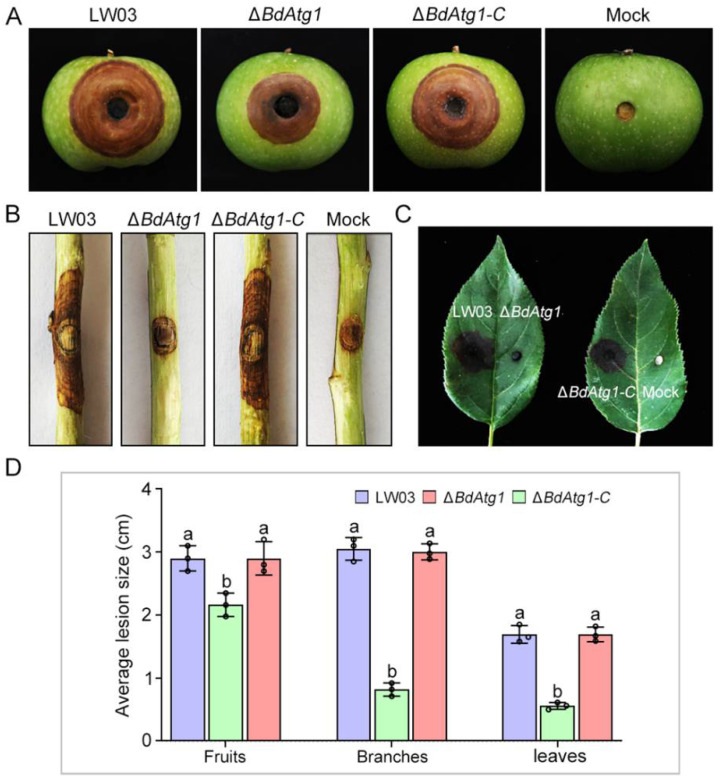
BdAtg1 is required for virulence. Disease symptoms on apple fruits (**A**), branches (**B**) and leaves (**C**) following inoculation with *B. dothidea* strains for 7 days. (**D**) Lesion size on apple fruits, branches and leaves caused by each strain. Error bars indicate standard deviation from three independent experiments. Values on the bars followed by the same letter are not significantly different at *p* = 0.05.

To determine the role of BdAtg1 in virulence, each strain was inoculated to young apple fruits, one-year-old branches and leaves. After 7 days of moisturizing incubation at 25 °C, the disease lesions caused by Δ*BdAtg1* were slightly smaller than that of LW03 and Δ*BdAtg1-C* (Figure 6A). Notably, Δ*BdAtg1* almost lost the ability to infect branches and leaves, while LW03 and Δ*BdAtg1-C* caused severe disease symptoms (Figure 6B–D). These results indicate that BdAtg1 plays a crucial role in plant infection in *B. dothidea*.

### 3.7. BdAtg1 Mediates Nuclei Distribution

**Figure 7:**
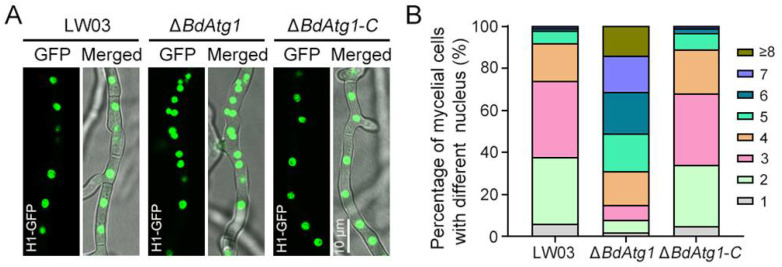
BdAtg1 mediates nuclei distribution. (**A**) Nuclei distribution in mycelia of *B. dothidea* strains. (**B**) The number of nuclei in mycelial compartment of each strain.

Autophagy has been reported to contribute to the regulation of nuclear dynamics in the filamentous fungus *Fusarium oxysporum* [21]. To test the effect of BdAtg1 on nuclear dynamic in *B. dothidea*, the nuclei in mycelial compartments of each strain was observed. As shown in Figure 7, the number of nuclei per mycelial compartment in LW03 and Δ*BdAtg1-C* was mostly 2 to 4, while the number of nuclei in Δ*BdAtg1* was mostly 4 to 8. These results indicate that BdAtg1 regulates nuclear dynamics in *B. dothidea*.

### 3.8. BdAtg1 Influences Multiple Metabolic Pathways

**Figure 8:**
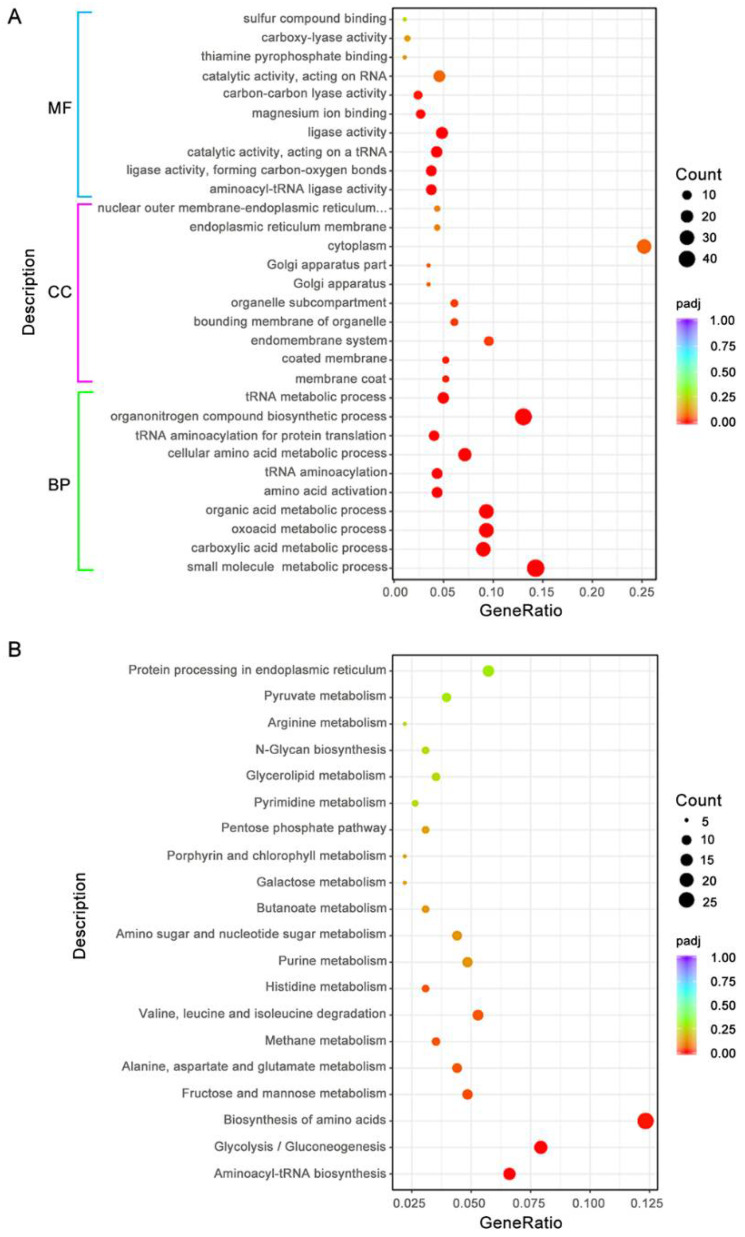
BdAtg1 influences multiple metabolic pathways. (**A**) Comparative Gene Ontology (GO) analysis of the differentially expressed genes (DEGs) between the transcriptome of LW03 and Δ*BdAtg1*. (**B**) Comparative KEGG pathways analysis of the DEGs between the transcriptome of LW03 and Δ*BdAtg1*.

Because of the number of severe phenotypic changes, we performed a transcriptome sequencing of LW03 and Δ*BdAtg1* during the mycelial growth stage. Compared with LW03, a total of 760 differentially expressed genes (DEGs) were identified in Δ*BdAtg1*, including 561 and 199 upregulated and downregulated genes, respectively (Figure S2). Gene Ontology (GO) and KEGG enrichment analysis revealed that inactivation of BdAtg1 significantly affected multiple metabolic pathways in *B. dothidea* (Figure 8). These results suggest that BdAtg1 regulates multiple metabolic pathways in *B. dothidea*.

## 4. Discussion

Autophagy is a catabolic process that maintains cellular homeostasis to ensure normal life activities in eukaryotes [22]. Recently, autophagy has been shown to play an important role in the growth, development and virulence in filamentous fungi [23,24]. In this study, we identified and characterized the autophagy protein Atg1 in *B. dothidea*. Phylogenetic tree and functional domain alignment suggested that BdAtg1 is evolutionarily conserved in eukaryotes. In addition, the deletion of *BdATG1* blocked the autophagy process in *B. dothidea*, which is consistent with that of *S. cerevisiae* and other filamentous fungi [25,26].

The endogenous recycling of intracellular components mediated by autophagy is crucial for material and energy supply during fungal vegetative growth and development [27]. In this study, the mycelial growth rate of Δ*BdAtg1* remained unchanged, but the amount of aerial mycelium was significantly reduced on media plates, which is consistent with the role of Atg1 in aerial mycelium growth in other filamentous fungi, such as *M. oryzae*, *B. cinerea* and *F. graminearum* [14,15,16]. In addition, Δ*BdAtg1* produced fewer conidia with malformation, displayed delayed conidial germination, and failed to form ascus, which is similar to the role of Atg1 in asexual and sexual reproduction in most filamentous fungi [23].

The infection process of pathogenic fungi requires autophagic degradation to meet the excessive demands for material and energy during this period [28]. Like other pathogenic fungi [29], the ability of Δ*BdAtg1* to infect apple fruits, branches and leaves was significantly reduced. Notably, Δ*BdAtg1* caused disease lesions on apple fruits, while almost losing the ability to infect branches and leaves. These findings suggest that BdAtg1 plays an important role in the infection process of *B. dothidea*.

To date, the biological function of autophagy has not been fully recognized in filamentous fungi. In this study, we analyzed the gene expression profiles through comparative transcriptome. The results revealed that the expression of many genes belonging to multiple metabolic pathways were affected in Δ*BdAtg1*. These results demonstrate the involvement of autophagy in multiple metabolic pathways in *B. dothidea*.

## 5. Conclusions

In summary, our results indicate that the autophagy protein Atg1 plays an important role in mycelial growth, conidiation, ascosporulation and virulence, and is involved in multiple metabolic pathways.

## Data Availability

All experimental data in this study will be made available upon reasonable request from readers.

## Supplementary Materials

The following supporting information can be downloaded at: https://www.mdpi.com/article/10.3390/jof8090904/s1, Figure S1: Target deletion of *BdATG1* in *B. dothidea*; Figure S2: Differentially expressed genes (DEGs) of transcriptome sequencing; Table S1: PCR primers used in this study.
