# Supplementary material for: Involvement of the Autophagy Protein Atg1 in Development and Virulence in Botryosphaeria dothidea
Source: J Fungi (Basel). 2022 Aug 26;8(9):904. doi: 10.3390/jof8090904 (PMC9501979; doi:10.3390/jof8090904)
Supplement: Supplementary file 1 [file jof-08-00904-s001.zip › jof-1838971-SI.pdf]

Table S1. PCR primers used in this study

Primer	Sequence (5′-3′)	Relevant characteristics
P1<br>P2	CGAGATCGCCAGGTCACGTG<br>CAAAATAGGCATTGATGTGTTGACCTCCGTGCGACGGGTGTGTTGGAG	PCR primers to amplify *BdATG1* upstream fragment for construction of the *BdATG1* deletion fragment
P3<br>P4	CTCGTCCGAGGGCAAAGGAATAGAGTAGCAGTTCAACCATGGCTCGTC<br>CGATCTTCCAGCACTCCGAG	PCR primers to amplify *BdATG1* downstream fragment for construction of the *BdATG1* deletion fragment
P5<br>P6	GCAGAGCGCTCGTATGTG<br>GCTGCAGGGTAACTAACTC	PCR primers to amplify the *BdATG1* deletion fragment for generation of the *BdATG1* deletion mutants
P7<br>P8	CCATCAACCTCGTCGCTGC<br>CCGTACCATGAACTGATGC	PCR primers for identification of *BdATG1* deletion mutants
Probe-F<br>Probe-R	GACAGGCAGACGTGCAGAC<br>GGCAGTGATGTGGGACATG	PCR primers to amplify the *BdATG1* upstream fragment used as the probe for Southern blotting analysis
ATG1-GFP-F<br>ATG1-GFP-R	ACTCACTATAGGGCGAATTGGGTACTCAAATTGGTTCGAACCGGTGATCTGAAC<br>CACCACCCCGGTGAACAGCTCCTCGCCCTTGCTCACGCGAGGCGATGCGTTGGC	PCR primers to amplify the native promoter and open reading frame of *BdATG1* to construct BdATG1-GFP fusion vector
ATG1-ID-F<br>GFP-ID-R	CGTATGCTTGAGGCAATC<br>GTGCTGCTTCATGTGGTC	PCR primers for PYF11-BdATG1-GFP vector identification
H1-GFP-F<br>H1-GFP-R	ACTCACTATAGGGCGAATTGGGTACTCAAATTGGTTGACAACACTCGATCAAGTC<br>CACCACCCCGGTGAACAGCTCCTCGCCCTTGCTCAGCCTTGGCCTCGGCCTC	PCR primers to amplify the native promoter and open reading frame of *BdH1* to construct BdH1-GFP fusion vector
H1-ID-F<br>GFP-ID-R	CTGCTGTACGTTTAGACG<br>GGCATGGCGGACTTGAAG	PCR primers for PYF11-BdH1-GFP vector identification

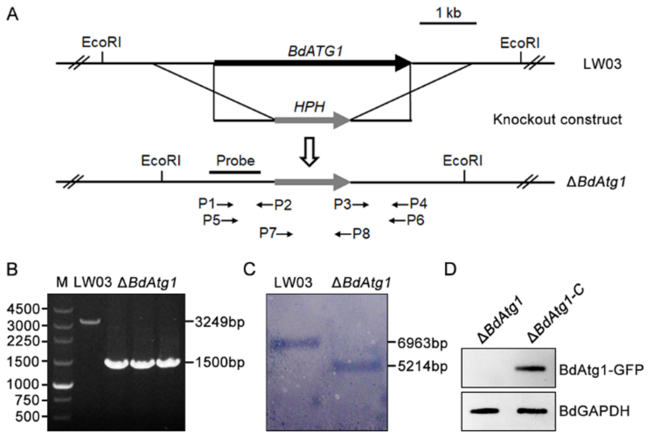

Figure S1: Target deletion of *BdATG1* in *B. dothidea*

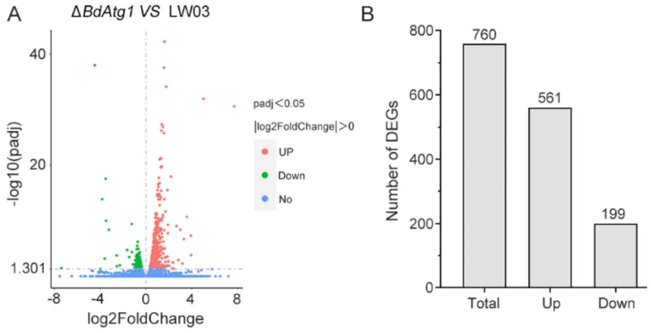

Figure S2: Differentially expressed genes (DEGs) of transcriptome sequencing
